# Spatial Modelling Tools to Integrate Public Health and Environmental Science, Illustrated with Infectious Cryptosporidiosis

**DOI:** 10.3390/ijerph13020186

**Published:** 2016-02-02

**Authors:** Aparna Lal

**Affiliations:** National Centre for Epidemiology and Population Health, Research School of Population Health, Australian National University, Acton, Canberra 2602, Australia; aparna.lal@anu.edu.au; Tel.: +61-2-61252309

**Keywords:** spatial, environmental change, climate, land use, disease

## Abstract

Contemporary spatial modelling tools can help examine how environmental exposures such as climate and land use together with socio-economic factors sustain infectious disease transmission in humans. Spatial methods can account for interactions across global and local scales, geographic clustering and continuity of the exposure surface, key characteristics of many environmental influences. Using cryptosporidiosis as an example, this review illustrates how, in resource rich settings, spatial tools have been used to inform targeted intervention strategies and forecast future disease risk with scenarios of environmental change. When used in conjunction with molecular studies, they have helped determine location-specific infection sources and environmental transmission pathways. There is considerable scope for such methods to be used to identify data/infrastructure gaps and establish a baseline of disease burden in resource-limited settings. Spatial methods can help integrate public health and environmental science by identifying the linkages between the physical and socio-economic environment and health outcomes. Understanding the environmental and social context for disease spread is important for assessing the public health implications of projected environmental change.

## 1. Environmental Change and Infectious Disease Spread

Global environmental changes, especially climate change and human exploitation of productive ecosystems have become increasingly important drivers of infectious disease spread [1,2]. For human pathogens with environmental reservoirs and transmission pathways, understanding geographical and seasonal variability in exposures can help to predict disease risk under scenarios of global environmental change [3]. New modelling tools are needed to identify the specific environmental exposures that aid spread and the socio-economic factors that sustain disease transmission. 

Regional variations in disease risk may be due to area-level characteristics as well as the characteristics of the individuals who live in these areas. Spatial approaches are useful for analyzing the associations between area-level environmental exposures, such as climate and proximity to livestock, and individual-level risk factors, such as age, susceptibility and disease risk. Studies that focus on individual risk factors only provide a snapshot of specific infection reservoirs and potential exposures. Further, they are not always financially viable. The increasing availability of large scale, environmental, socio-economic and demographic data sources and freely available software makes an integrated approach using spatial methods possible [4].

## 2. The Utility of Spatial Methods to Assess the Public Health Impacts of Environmental Change

Spatial models have become an important tool to examine scale-specific influences. While changes in climate and livestock production practices can drive disease emergence at a global scale, socio-economic factors are more likely to influence disease spread locally. Spatial models explicitly take into account such hierarchical structure in the data. Such models have frequently been applied to model the distribution of vector-borne diseases such as, dengue and malaria [5,6], but are less common for other environmentally transmitted diseases spread from animals to humans. Spatial models can partition out the impact of environmental, socio-economic and demographic variables and individual level risk factors to quantify their relative contribution to disease incidence. 

Geographical location can influence the risk of infection and confound the association between disease risk and environmental factors. Areas characterized by some populations (e.g., those of lower income and education) and by some location characteristics (city dwelling *vs.* remote) may also to be characterized by higher exposures to environmental risk factors. Examining disease patterns after modelling out the clustering due to location (spatial autocorrelation) can help to identify which factors underlie the observed disease patterns. Moran’s I and the inversely related Geary’s C [7] are frequently used indicators to examine whether disease outcomes are spatially clustered, randomly or uniformly distributed in space. Another tool to measure spatial dependency is the semi-variogram [8], which illustrates the range and the rate at which spatial autocorrelation declines. In a dataset with spatial autocorrelation, the semi-variance would increase to a maximum value before levelling off. Initial analyses using these descriptive tools can provide important insights into the spatial nature of disease spread.

Environmental exposures such as climate and land use are spatially dependent (*i.e.*, individuals closer together are more likely to have a similar exposure than individuals further apart). In such instances, analyses that do not account for spatial dependence in exposures may lead to overstating the significance of the results. Non-spatial methods applied to such data typically give smaller standard errors for point estimates, compared to spatial models [9], potentially leading to misplaced confidence in the association. In this respect, spatial models are a substantial improvement over non-spatial models. For example, a global analysis of waterborne disease outbreaks identified that population density and accumulated temperature as significant risk factors [10]. Importantly, although the point estimates for the effects of these factors were similar for spatial and non-spatial models, after controlling for the spatial correlation structure in the data, the spatial models were a better fit for the data [10]. 

Cluster detection is a widely used technique to identify areas of high disease risk. However, a limitation of area based space-time cluster analyses such as those implemented in SatScan [11] is that the heterogeneity and changing trends of the underlying population is usually not accounted for. In such cases, emerging hotspots may just represent spatial population mobility patterns. Moreover, for environmental exposures in particular that represent a continuous exposure surface rather than an exposure that stops at a pre-determined boundary or has a defined shape, such approaches are less robust. A recent extension of cluster detection methods that allows the spatial autocorrelation structure and the continuity of the exposure to be modelled is the exceedance probability in a Bayesian framework. This approach identifies areas where the probability of the relative risk of disease would exceed a specified threshold. While this technique is dependent on the precision of relative risk estimates, it offers a more flexible and conservative approach to spatial cluster detection and is particularly useful to model environmental exposures [12,13].

## 3. Spatial Epidemiological Approaches: Challenges and Opportunities

### 3.1. Challenges

As with any statistical approach, the research question and the available data should drive the choice of analytic methods. The ability to link data by location underpins spatial analytical methods. However, these data intensive methods have limitations that need to be understood to aid valid use and interpretation. First, the lack of data at an appropriate geographic scale is potentially one reason for the few spatial studies on cryptosporidiosis. Locally relevant environmental exposures such as livestock information are often hard to obtain, limiting the possibility of such analyses. However, the increasing availability of open access data sources such as the Gridded Livestock of the World database [14] and the IRI Climate Data Library [15] at a locally appropriate scale offer promising avenues for future research in this area. Second, the trade-off between high spatial resolution analyses and patient privacy is a challenge unique to spatial models [16]. A recently developed method allows separation of physical address and clinical information, preserving the ability to conduct high precision spatial analyses using individual patient records without loss of patient privacy [17]. Third, spatial methods are computationally more intensive and require expertise and an understanding of how to formulate spatial models. However, the increasing capacity of computers and popularity of machine learning approaches using open access software is rapidly minimizing this problem. Fourth, as spatial approaches link human illness data to exposure data using location it is difficult to prove causation from such observational studies. As recently shown by Cox *et al*. [18] health outcomes may be significantly statistically associated with environmental exposures due to spatial location, which can act as a confounder when there are spatial patterns in both exposure and response variables. However, as discussed, there are techniques to filter out the underlying spatial patterns in explanatory and outcome variables to examine and infer causal associations in such instances [18]. Fifth, a major consideration when interpreting spatial exposure—response associations is that population—level associations cannot be assumed at the individual-level, due to considerable person-level heterogeneity [19]. This “ecological fallacy” [20] can lead to erroneous effect attribution. One approach would be to estimate the effect of environmental exposures on individual health outcomes, if individual level outcomes are of interest [21]. However, equally important to note is that wider socio-ecological environment sets the context for disease transmission and maintenance in a population [22] and is key to determine exposure and susceptibility to infection [23,24]. For environmental exposures like weather and climate, in some instances, it is the higher level ecological associations that can better capture the complexity of the relationships, compared to locality specific measures [25].

### 3.2. Opportunities

It is important to recognize the opportunities created by spatial epidemiological approaches. First, in low-income countries, infectious diseases already have a considerable social and economic impact [26,27,28]. Current trajectories of climate variability and intensified livestock production have the potential to worsen this situation [29,30]. Thus, in regions that lack the necessary resources for disease control and prevention, environmental changes could become a dominant driver for some infectious diseases. In such areas, spatial methods can help to establish a baseline burden of disease. For example, the sparse and disparate data available on the global distribution of the environmentally sensitive leishmaniases prompted the compilation of a global, geographical database to generate high-resolution global distribution risk maps for the leishmaniases, providing an important baseline assessment [31]. Such information can considerably improve the accuracy of global estimates of infectious disease burden [32]. Second, spatial methods allow data collected for different purposes such as census and meteorological data to be linked to disease data through the common attribute of location. These methods provide a platform to address the widely acknowledged need for greater integration of disciplines to examine complex public health issues [33]. India, with its repository of detailed climate and health associated data has been identified as a key player in developing and promoting climate health research in emerging economies [34]. Third, spatial methods offer an opportunity for timely and resource efficient disease surveillance in countries without conventional disease surveillance systems [35]. For example, a spatial approach to disease control has resulted in a significant decrease in schistosomiasis in China, following the rapid dissemination of epidemiological information allowing a targeted public health response [36]. Fourth, spatial tools can help identify the locality specific climate signals that precede disease outbreaks as environmental conditions are a key factor that can limit or facilitate the spread of many infectious pathogens. Locality and season specific dengue epidemics have been successfully forecast using climate predictions in Brazil [5]. This information can help maximize the lead time in early warning systems, where changing environmental and social factors can be used to predict the risk of outbreaks in real time [37]. In summary, spatial methods provide important insights into the determinants of disease risk in areas with limited data and where public health agencies do not have the infrastructure or resources to implement traditional surveillance. These methods are especially suited to proactively monitor changes in disease risk as a result of changing environmental conditions.

## 4. Cryptosporidiosis: A Case Study

Infectious diarrhoea, caused by the parasite *Cryptosporidium,* has been recognized by the World Health Organization as a public health infection of global importance [38]. The parasite is resilient to a wide range of environmental conditions and chemicals and is the most commonly isolated parasite in domestic animals [39]. It is spread through the faecal–oral route; either through direct contact with an infected person or animal or, indirectly, through contaminated food, water and environments. These characteristics facilitate parasite spread, making cryptosporidiosis a challenge to control. 

Infection is common in developed countries, where children <5 years old bear the greatest burden of cryptosporidiosis [40,41,42]. Positive area-level associations of disease with livestock density have been reported from New Zealand [43], the US [44] and Scotland [45], and in rural Canada [46]. There are clear differences in seasonal patterns of cryptosporidiosis across urban-rural areas and molecular evidence suggests that there is considerable potential for zoonotic transmission from cattle in rural regions [46,47]. For example, in Scotland, *Cryptosporidium* hominis (human strain) cases were associated with higher population density, while high ruminant density and rural living were associated with increased risk in *C*. *parvum* (livestock strain) cases [48]. Similar spatially distinct patterns in *Cryptosporidium* strains have been reported from England and Wales [49] and New Zealand [50,51]. In areas with adequate resources, spatial methods provide impetus for targeted resource intensive molecular studies [52,53,54] and for cryptosporidiosis, have consistently shown that rural areas, dominated by cattle are hotspots for zoonotic transmission of the disease.

Climate change is likely to influence cryptosporidiosis incidence patterns, as the parasite is easily transmitted through the environment, particularly through water. Historical time-series modelling of cryptosporidiosis incidence with rainfall have reported positive associations in a global meta-analysis of cryptosporidiosis seasonality [55]. High rainfall may facilitate pathogen runoff, contaminating surface and drinking water sources [56]. Although increased runoff is an inevitable consequence of increased rainfall, increased runoff may also follow extended dry periods. Low water flows may promote pathogen concentration, leading to a higher pathogen load, resulting in increased disease. In New Zealand, a spatial study of cryptosporidiosis patterns showed that the quality of public drinking water supplies modified the effect of rainfall [57]. A 36% increase in reported cryptosporidiosis is estimated with future climate change projections in New Zealand, with children most at-risk [58]. Increased investment in drinking water treatment and management during extreme rainfall events may help reduce waterborne cryptosporidiosis. 

Socio-economic disadvantage may amplify disease risk in some populations. For example, cryptosporidiosis incidence in Brisbane, Australia was positively associated with the proportion of residents with low education attainment, after correcting for spatial dependency [59]. A recent descriptive study of cryptosporidiosis across Australia found that the highest rates of disease were concentrated in the tropical, remote parts of the country [60], with a significant association of disease risk with increasing remoteness shown for children less than 5 years old [61]. Many of these areas tend to be characterized by less regulated drinking water supplies, lower levels of income and increased household crowding, known risk factors for cryptosporidiosis. An increased public health focus with respect to interventions for disease control in needed to reduce cryptosporidiosis in these regions. 

In low income countries, cryptosporidiosis is associated with an increased risk of death in children between 1–2 years of age, as estimated by the recent Global Enteric Multi-Centre Study [62]. A recent review found that *Cryptosporidium* spp. was significantly more prevalent in malnourished children 2–5 years old with diarrhoea compared to non-malnourished children [63]. Despite this, there is little recognition of the importance of this disease [64] and no systematic approach to understand how environmental exposures and socio-economic factors contribute to disease spread in these regions with disproportionately high disease rates [65]. As has been done for leishmaniases, a useful starting point would be to utilise disease incidence data available from multicounty studies of cryptosporidiosis [62] to produce global risk maps. Such initial analyses when combined with freely available global datasets on climatic variables can provide important information on environmental risk factors in in resource-constrained settings. For example, in Zambia, the peak in *Cryptosporidium* infections at the start of the rainy season [66] has been attributed to renewed watering holes serving as a playing area for children and water source for animals, with increased opportunities for zoonotic transmission [67]. In India, geographic clustering of genetically similar strains of cryptosporidiosis [68] and the first multisite study of the disease [69] show that while spatial methods to understand cryptosporidiosis epidemiology in such settings is still in its infancy, there is immense scope for such analysis.

## 5. Conclusions

Well-designed spatial statistical models can aid our understanding of how wider socio-environmental interactions may mediate infectious disease spread and account for disparities in disease risk. A significant opportunity exists in using open access spatial environmental and socio-economic data to examine these relationships in resource poor settings, where disease burden is disproportionately high. Knowledge gained could strengthen global public health surveillance and help develop interventions for disease prevention, with future environmental change. 
